# Effects of dexamethasone on angiotensin II-induced changes of monolayer permeability and F-actin distribution in glomerular endothelial cells

**DOI:** 10.3892/etm.2013.1278

**Published:** 2013-08-30

**Authors:** JUNYAN FANG, MIAO WANG, WEI ZHANG, YINGDENG WANG

**Affiliations:** Department of Clinical Nephrology, Shanghai Ninth People’s Hospital, Shanghai Jiao Tong University School of Medicine, Shanghai 200011, P.R. China

**Keywords:** angiotensin II, dexamethasone, endothelial cells, F-actin, injury, monolayer permeability

## Abstract

The aim of this study was to investigate the changes in monolayer permeability and F-actin distribution caused by angiotensin II (Ang II)-induced injury in glomerular endothelial cells (GENCs) and the effects of dexamethasone on these changes. GENCs isolated and cultured from Wistar rats were used to examine the changes in monolayer permeability and F-actin distribution induced by Ang II. GENC permeability was evaluated by measuring the diffusion of biotin-conjugated bovine serum albumin (biotin-BSA) across a cell monolayer. The expression levels and distribution of F-actin were assessed by flow cytometry. The biotin-BSA concentrations were measured by capture enzyme-linked immunosorbent assay. Ang II at a concentration of 10 mg/l increased the permeability of the GENC monolayer at 6 h and 12 h (P<0.05 and P<0.01, respectively) and caused F-actin depolymerisation at 6 h and 12 h (P<0.01). The two effects attributed to Ang II were significantly inhibited by dexamethasone treatment (P<0.01). The increased permeability of the GENC monolayer induced by Ang II was significantly correlated with the depolymerisation of F-actin. Dexamethasone abrogated the Ang II-mediated damage to GENCs indicating that it may play an important role in protecting GENCs from injury.

## Introduction

The development and progression of chronic kidney disease (CKD) is associated with inflammatory responses of various etiologies (1,2). Several previous studies have demonstrated that a variety of inflammatory mediators are involved in the pathophysiological processes of CKD (3,4). Glomerular endothelial cells (GENCs), which are important components of the glomerular filtration barrier, are the main target cells for inflammatory mediators and are important in the initiation and progression of CKD (5,6). Several studies have shown that changes in the structure, distribution and function of the endothelial cell skeleton are the main mechanisms underlying the increased vascular permeability during early inflammation (7). F-actin, the main component of the cytoskeleton, is rearranged to enable endothelial cell (EC) contraction, crack formation and increases in permeability when regulated by various inflammatory mediators (8). Angiotensin II (Ang II) is the major bioactive substance in the renin-angiotensin system (RAS). It is involved in the regulation of vascular tension and blood flow, and the promotion of cell growth and proliferation, and may also act as a proinflammatory factor. Studies have confirmed that the activity of the RAS in the kidney tissues of patients with CKD is elevated independently of the presence or absence of hypertension, and that the concentration of Ang II is significantly higher than that in plasma, indicating its importance in inflammation-mediated EC injury (9,10). The aim of the present study was to investigate the mechanism by which Ang II causes inflammatory damage in GENCs by observing the effects of Ang II on GENC monolayer permeability and F-actin distribution.

## Materials and methods

### Reagents and animals

Dulbecco’s modified Eagle’s medium (DMEM) dried powder and fetal bovine serum (FBS) were purchased from Hyclone (Logan, UT, USA). Fluorescein isothiocyanate (FITC)-phalloidin and trypsin (activity, 1:20) were purchased from Sigma (St. Louis, MO, USA). Sumianxin was provided by the Veterinary Research Institute of Changchun University (Changchun, China). The VIII R:Ag testing kits (secondary antibodies tagged with biotin; DAKO, Carpinteria, CA, USA), CD31 and CD34 were purchased from Dako (Carpinteria, CA, USA). Heparin was purchased from the Nanjing biochemistry pharmaceutical factory (Nanjing, China). The nitrate-mixed acetate fibre membrane (0.45 *μ*m) was provided by the Shanghai New Asia Purification Devices Plant (Shanghai, China).

This study was performed at the Shanghai Ninth People’s Hospital affiliated with Shanghai Jiao Tong University School of Medicine (Shanghai, China) from October 2010 to November 2011. All animal experimental protocols were approved by the Animal Care and Use Committee of Shanghai Ninth People’s Hospital affiliated with Shanghai Jiao Tong University School of Medicine and conformed to the Guide for the Care and Use of Laboratory Animals (National Research Council, Chinese version, 1996) (11). A total of 25 male Wistar rats (weight, ~120 g) were supplied by the Shanghai Experimental Animal Center of Chinese Science College (Shanghai, China) and housed in a room (n=3 rats per cage) with a controlled temperature and humidity. The rats were fed with standard rat chow and had access to tap water *ad libitum*.

### GENC isolation, culture and identification

Following a previously published protocol (12,13), the 25 male Wistar rats were anaesthetized by intraperitoneal (i.p) injection of sumianxin (0.8 mg/kg) and received an i.p injection of 3,000 units heparin sodium. After being fixed in a supine position, the chest and abdomen of each rat were disinfected and the thoracic aorta was isolated for perfusion. Cold aseptic Hank’s solution was used to wash away the blood while the renal vein was cut to be conducive to the liquid outflow. After 1–2 min, when the color of the kidneys appeared white, the kidneys were placed into an ice-bath for preservation. Under sterile conditions, the renal capsule was torn from the lavaged kidney and the renal cortex was cut into 1–2-mm^3^ thick fragments. The kidney fragments were ground with a 100 mesh steel sieve and then successively filtered through 150 and 200 mesh steel sieves, respectively. The resultant glomeruli were collected in the 200 mesh steel sieve. Microscopic observations identified that the purity of the glomeruli was 99%. Following centrifugation at 462 × g for 5 min, the glomeruli were washed twice with serum-free DMEM. The separated glomeruli were digested with type IV collagenase, mixed with DMEM and then centrifuged at 462 × g for 5 min. The supernatant was collected and centrifuged again at 462 × g for 5 min to provide a precipitate containing the ECs. Approximately 10^2^–10^3^ primary cells were isolated from each kidney resulting in a passage proportion of 1:2. The glomerular ECs were seeded in a germ-free plastic bottle pre-coated with 1% gelatin and the prepared EC cultured medium (DMEM medium with 20% FBS, 5 U/ml heparin, 5 U/l insulin, 5 *μ*g/ml transferrin and 5 *μ*g/ml selenium) was added. The cells were cultured in an incubator at 37°C with 5% CO_2_ and a humidity of 95–100%. The cultured medium was replaced every 3–4 days until 80–90% of the cells formed a confluent monolayer in the 2nd–3rd week. The cells were digested with 0.25% trypsin and then subcloned in 96-well plates at different densities as follows: 1 cell per well in the 1st row, 2 cells per well in the 2nd row, 4 cells per well in the 3rd row, 8 cells per well in the 4th row, 16 cells per well in the 5th row, 32 cells per well in the 6th row, 64 cells per well in the 7th row, and 128 cells per well in the 8th row. When cloning could be observed after culturing for 3–4 days, the cells were collected into culture bottles for further culturing. The cultured medium was changed every 3–4 days and the cells were subcultured every 6–7 days. The cells were digested with trypsin, and digestion was terminated by the addition of DMEM with 10% FBS. The cells were cultured in separate bottles at a ratio of 1:2. Cells in the 2nd–3rd generations were identified by detecting factor VIII-associated antigen, CD31 and CD34, and morphological observations. The 2nd–3rd generation cells were used during this experiment as they did not show cell senescence, differentiation or cytometaplasia during repeated passages (14,15).

### Detection of the effects of Ang II on F-actin by flow cytometry

GENCs were cultured in 50 ml culture bottles; Ang II (10 mg/l) and Ang II (10 mg/l) + dexamethasone (10 mg/l) were separately added once 80–90% of the cells had formed a confluent monolayer. In addition a normal control group (GENC with FITC-phalloidin) and a negative control group (GENC without FITC-phalloidin) were established. Cells were removed from the culture bottles after 6 and 12 h and treated as follows: (i) The cells were washed twice with DMEM containing 1% FBS; (ii) the cells were digested with 0.5% trypsin and placed into a flow cytometry detection tube; (iii) the cell suspension was centrifuged at 1,847 × g for 10 min and the supernatant was discarded; (iv) the precipitate was washed twice with phosphate-buffered saline (PBS) and centrifuged at 462 × g for 5 min to isolate the cells; (v) the cells were fixed with 4% paraformaldehyde for 10 min at 4°C and 30 min at room temperature and the samples were then centrifuged at 462 × g for 5 min to remove the supernatant; (vi) the cells were washed twice with PBS and centrifuged at 462 × g for 5 min to discard the liquid; (vii) 1 ml of 0.5mg/l FITC-phalloidin (100 *μ*g FITC-phalloidin was dissolved in 0.2 ml methanol and diluted with 0.01 M PBS) was added, and the cells were reacted for 40 min in the dark; (viii) samples were washed with PBS three times and centrifuged at 462 × g for 5 min to remove the supernatant and eliminate the uncombined FITC-phalloidin; and (ix) cells were gently blended after adding 0.8 ml PBS. The changes in F-actin were detected by flow cytometry (FACScalibur™; BD Biosciences, Franklin Lakes, NJ, USA), with the absence of FITC-phalloidin in the negative control group (16,17).

### Detection of EC growth on the filter membrane

The filter membrane was treated with 0.5% acetic acid for 20 min at 50°C, boiled for 60 min in 0.1% gelatin and then parched with fire prior to use. GENCs were inoculated on the membrane at a density of 5×10^5^/per hole. The membrane was removed from the nesting after 7–10 days, based on previous studies (17,18), which showed that ECs reach a state of cell fusion and covered the filter membrane within a period of 7–10 days. The membrane was then washed three times with PBS, fixed with 95% alcohol for 10 min, washed again three times with PBS, stained with hematoxylin and eosin for 5 min and rinsed with water three times. Furthermore, the membrane was weathered with 1% hydrochloric acid alcohol and dyed blue with 1% ammonia. The cells were monitored by transmission light microscopy, and detection was performed when 80–90% of the cells became a converged monolayer. One random nested filter membrane was dyed prior to detection to determine whether the ECs on the filter membrane reached a condition of cell fusion. In addition, all nested filter membranes were dyed at the end of the experiment to test the EC integrity.

### Permeability test

The permeability test was performed using a modification of a previously published method (17,18). Briefly, GENCs were digested with 0.5% trypsin to prepare a cell suspension and the cell count was adjusted to 3.3×10^5^/ml. The cell suspension (1.5 ml) was inoculated on the handled membrane in the micropore nest for culturing the cells, which was placed into a small well of a 6-well culture plate with 2.5 ml culture medium in each well. The plate was placed in an incubator with 5% CO_2_ at 37°C and 95–100% humidity and the culture medium was replaced every 2 days. The nest and the small well of the 6-well plate consisted of two relatively isolated chambers (the inner and outer chambers). The exchange of substances between the inner and outer chambers was mediated by the filter membrane and the EC mono-layer. When the ECs reached a certain level of confluency, 10 mg/l Ang II diluted in DMEM containing 1% FBS (2 ml per nest) was added in one group, and 10 mg/l Ang II + 10 mg/l dexamethasone (2 ml) was added to the remaining group; the control group was treated with 2 ml DMEM containing 1% FBS. Simultaneously, 100 mg/l biotin-BSA was added as a permeability indicator. The cultured media of the inner and outer chambers were collected separately after 6 and 12 h to detect the concentration of biotin-BSA (n=4 per group). The albumin clearance of the EC monolayer was calculated with the following formula: Clearance (%) = biotin-BSA density of outside chamber/biotin-BSA density of inner chamber ×100.

### Biotin-BSA density test

The capture enzyme-linked immunosorbent assay (ELISA) was used to detect the concentration of biotin-BSA in the inner and outer chambers (Vector Laboratories, Burlingame, CA, USA). A micropore plate was coated overnight with 5 mg/l streptavidin at 4°C. The liquid coating was removed, 300 *μ*l/well blocking buffer was added and the plate was incubated at room temperature for 2 h and then washed three times with PBS. After diluting a 1-ml sample at a ratio of 1:100, the sample was added to the ELISA plate to react for 1 h at room temperature. The plate was washed three times with PBS and 100 *μ*l streptavidin conjugated with 5 mg/l horseradish peroxide enzyme was added to react for 1 h at room temperature. Again, the plate was washed six times with PBS. Tetramethyl-benzidine was added as the bottom material and coloration was allowed to develop for 20 min at room temperature. Finally, 1.8 mol/l H_2_SO_4_ (80 *μ*l) was added into each well to inhibit the reaction and the absorbance at 450 nm was measured in the microplate.

### Statistical analysis

Data were analyzed with SPSS software, version 11.0 (SPSS, Inc., Chicago, IL, USA). The results are expressed as the mean ± standard deviation (19). Differences between groups were assessed using the t-test and one-way analysis of variance, and the Spearman grade correlativity was used to verify the correlation between the GENC monolayer permeability and distribution of F-actin. P<0.05 was considered to indicate a statistically significant difference.

## Results

### GENC morphology, growth characteristics and identification

**Figure 1.**
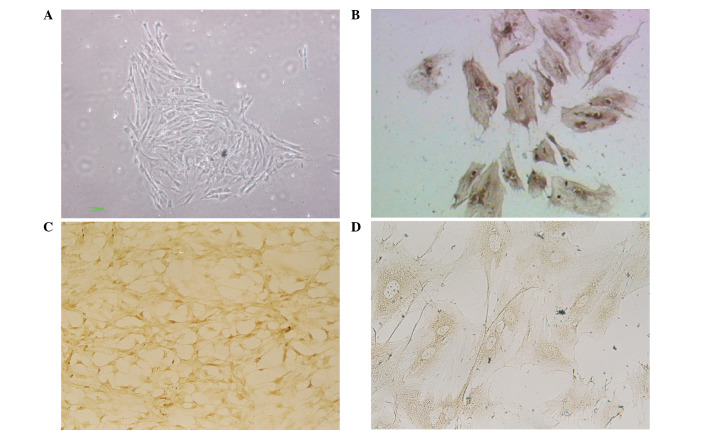
GENC morphology, growth characteristics and identification. (A) Normal control group after 6 days of primary culture (inverted microscope; magnification, ×100); positive staining of (B) GENC VIII-associated antigen (immunohistochemistry image; magnification, ×200), (C) GENC CD31 (immunohistochemistry image; magnification, ×200) and (D) GENC CD34 (immunohistochemistry image; magnification, ×200) were observed. GENC, glomerular endothelial cell.

When observed under an inverted microscope, the GENCs appeared adherent following primary culture for 48 h and began to grow and divide after 72 h. The majority of the cells were round and polygonal and showed a tendency to form a glomus. Evident nuclei and a small number of cells were interconnected with each other as observed under a high magnification (inverted microscope; magnification, ×100). Monolayer convergence was identified at 2–3 weeks, at which time the majority of cells showed a polygonal and short fusiform morphology with an appearance similar to that of paving stones. Cells in the split-phase appeared quasi-circular and marginally stained showing single-layer adherent growth. Contact inhibition occurred among the cells as detected by the arrest of cell division when the single-layer was completely convergent (Fig. 1A). The GENCs were positive for factor VIII-associated antigen, CD31 and CD34 reflecting the endothelial growth characteristics of these cells (Fig. 1B–D).

### Effects of Ang II on GENC monolayer permeability

The albumin permeability of GENCs treated with 10 mg/l Ang II increased significantly following 6 and 12 h of culture compared with that of the untreated control group (P<0.05 and P<0.01, respectively). The albumin permeability of GENCs treated with 10 mg/l Ang II and dexamethasone was significantly lower than that of cells treated with Ang II alone (P<0.01) indicating that dexamethasone inhibited the Ang II-induced increase of albumin permeability and played a protective role (Table I).

### Effects of Ang II on GENC F-actin detected by flow cytometry

**Figure 2.**
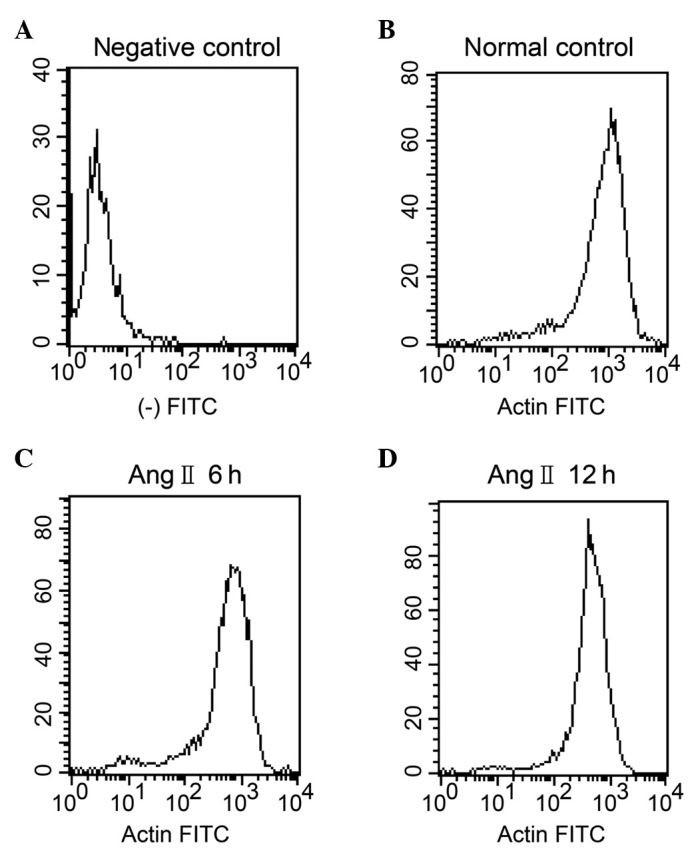
Effects of Ang II on F-actin levels in GENCs detected by flow cytometry at different times in the (A) negative control group, (B) normal control group, (C) Ang II 6 h group and (D) Ang II 12 h group. Ang II, angiotensin II; GENC, glomerular endothelial cell; FITC, fluorescein isothiocyanate.

The fluorescence intensity of F-actin in the GENCs treated with 10 mg/l Ang II decreased significantly after 6 and 12 h of culture compared with that of the normal control cells at the corresponding times (P<0.01). The fluorescence intensity of F-actin in GENCs treated with Ang II and dexamethasone was significantly higher than that of the cells treated with Ang II alone (P<0.01) indicating that dexamethasone inhibited the depolymerisation of F-actin induced by Ang II and played a protective role (Table II, Fig. 2). FITC-phalloidin was not detected in the negative control group.

### Correlation between GENC monolayer permeability and F-actin

The Spearman grade correlation coefficient was used to examine the correlation between the GENC monolayer permeability and F-actin distribution. The results showed a negative correlation indicating that monolayer permeability increased with F-actin depolymerisation (r=−0.901, P<0.01).

## Discussion

The vascular endothelium, including the EC monolayer and basement membrane, is a semi-selective permeability barrier that lines the luminal surface of blood vessels. The exchange of solutes and liquids between the inner and outer surfaces of blood vessels is regulated by the vascular endothelium. Permeability is an objective measurable indicator of endothelial barrier function. Glomerular endothelial cells are important for the integrity of the glomerular vascular structure and are also an active organ with autocrine and paracrine secretion functions. Endothelial cell dysfunction and decreased endothelial cell concentration are significant in the development of progressive renal disease and chronic renal failure (20). Increased glomerular filtration barrier permeability is a pathological feature and a critical factor in the etiology of CKD. Therefore, cultured GENCs are critical for studying the regulatory mechanisms of glomerular filtration barrier permeability, which is of great significance for the identification of effective treatments to prevent and cure CKD.

The mechanisms underlying the increased permeability of ECs caused by various damaging factors may be broadly classified into two categories (21): (i) In cases of severe injury or delayed response time, EC dissolution or detachment from the basement membrane occurs, which affects the integrity of the EC monolayer; and (ii) damaging factors cause EC contraction or retraction, resulting in the formation of cracks between cells. In recent years, the latter mechanism has received increased attention. In a previous study the enhanced microvascular permeability observed was mainly due to the retraction of microvesicular ECs causing an increase in the space between cells and EC injury (21). In response to inflammatory mediators, such as lipopolysaccharides and platelet activating factor, F-actin, an essential component of the endothelial cytoskeleton, is depolymerised and rearranged to increase tension, resulting in an intense cell contraction. Furthermore, F-actin may affect the function of tight and adherent junctions, damaging the integrity of the ECs, which results in the formation of EC gaps that increase permeability (22,23).

In the present study, we observed that normal rat GENCs showed a polygonal and short fusiform morphology *in vitro*, in a pattern similar to that of paving stones. The cells in split-phase were quasi-circular with a light color and showed single-layer adherent growth. The GENCs were positive for factor VIII-associated antigen, CD31 and CD34, which confirmed their endothelial cell growth characteristics. Ang II is the major bioactive substance in the RAS, and is associated with the regulation of vascular tension and blood flow, promotes cell growth and proliferation, and acts as a proinflammatory factor. Ang II binds to a receptor of the G protein-coupled receptor (GPCR) family and regulates a variety of cytokines and inflammatory mediators to induce inflammatory cell activation, which triggers the inflammatory cascade (22,24). Inflammatory cells may also activate the RAS, which enhances the effects of Ang II on local inflammation and in the process of inflammation (21). Studies have confirmed that RAS activity in the kidney tissues of patients with CKD increases regardless of the presence or absence of hypertension, and its concentration is significantly higher than that in the plasma (10). In the present study, we showed that F-actin was depolymerised in GENCs treated with 10 mg/l Ang II for 6 and 12 h. Furthermore, we examined the changes in the monolayer permeability of GENCs in response to Ang II treatment at different time points and identified that monolayer permeability increased at 6 and 12 h. These results indicated that Ang II induced F-actin depolymerisation and increased monolayer permeability of GENCs at a certain density. This increase in monolayer permeability may be negatively correlated with F-actin depolymerisation. The increase in monolayer permeability of Ang II-treated GENCs at 6 and 12 h was followed by fracture at 24 h. These results indicated that Ang II may damage the barrier function of ECs by combining with a specific receptor, such as a GPCR, to activate a G protein function and further activate phospholipases (PL)-D, PLC, PLA_2_ and the Ca^2+^ channel. PLC produces DAG and activates protein kinase C (PKC). PKC activation, Ca^2+^ and Ca^2+^/CaM induce the myosin light chain kinase, which mediates the phosphorylation of the myosin light chain, promotes F-actin depolymerisation, induces the interaction between actin and myosin, and affects cell adhesion connections and tense connections, resulting in the formation of EC gaps and increased permeability (25,26). The mechanisms of Ang II may be as follows: (i) Ang II may exert direct effects on GENCs and a low concentration of Ang II may alter the cellular morphology and induce actin depolymerisation, rearrangement and fibrin loss, thereby increasing the mono-layer permeability; and (ii) Ang II may stimulate GENCs to produce inflammatory mediators, such as IL-6, IL-8 and NF-κB, to result in cell injury (27).

Hormones are a major drug type in the treatment of kidney diseases and their anti-inflammatory, anti-immunisation and antitoxin effects have been demonstrated previously (28,29). In the present study, we observed inhibition of the Ang II-induced increase in monolayer permeability and F-actin depolymerisation when dexamethasone was used in combination with Ang II. This result indicated that dexamethasone may have inhibited the inflammatory factors, such as IL-6, IL-8 and NF-κB, that were induced by Ang II and thereby protect the GENCs. Our results partly clarified the mechanisms underlying the inflammatory injury of the GENCs caused by Ang II, which may provide a theoretical basis for the design of therapeutic strategies against CKD development and progression.

## Figures and Tables

**Table I.** Effect of Ang II on GENC monolayer permeability.

Group (n=3)	Monolayer clearance (%)	

	6 h	12 h
Filter without ECs	94.65±0.42	99.62±0.71
Normal control	27.71±0.21	27.78±0.48
Ang II	32.97±0.91^a^	34.83±1.2^b^
Ang II + dexamethasone	29.16±0.36^c^	28.03±0.46^c^

a P<0.05 and

b P<0.01 compared with the normal control group;

c P<0.01 compared with the Ang II group. Ang II, angiotensin II; GENC, glomerular endothelial cell; EC, endothelial cell.

**Table II.** Effects of 10 mg/l Ang II on GENC F-actin levels.

Group (n=4)	Fluorescence intensity of F-actin in GENCs					
	Negative control	Normal control	Ang II 6 h	Ang II 12 h	Ang II 6 h + dexamethasone	Ang II 12 h + dexamethasone
Fluorescence intensity	3.095±0.320	965.40±17.74	573.69±23.99^a^	326.95±2.53^a^	770.12±10.95^b^	650.36±23.78^b^

a P<0.01 compared with the normal control group and

b P<0.01 compared with the corresponding Ang II group. No fluorescein isothiocyanate (FITC)-phalloidin was observed in the negative control group. GENC, glomerular endothelial cell; Ang II, angiotensin II.
